# RNA‐Seq analysis reveals the different mechanisms triggered by bovine and equine after infection with FMDV

**DOI:** 10.1002/vms3.1569

**Published:** 2024-09-17

**Authors:** Yi Wu, Lu Li, Wanfu Bai, Tao Li, Xiaoying Qian, Yiyi Liu, Shenyuan Wang, Chunxia Liu, Fang Wan, Dong Zhang, Yingchun Liu, Kaifeng Wu, Yu Ling, Huanmin Zhou, Fanhua Meng, Yanru Zhang, Junwei Cao

**Affiliations:** ^1^ College of Life Sciences Inner Mongolia Agricultural University Hohhot China; ^2^ Inner Mongolia Key Laboratory of Biomanufacturing Hohhot China; ^3^ Inner Mongolia Endemic Livestock Biotechnology Innovation Team Hohhot China; ^4^ BaoTou Medical College Baotou China; ^5^ Institute of Agriculture and Animal Husbandry Xing'an League China; ^6^ School of Medicine Hainan Vocational University of Science and Technology Haikou China

**Keywords:** apoptosis, autophagy, FMDV, RNA‐Seq

## Abstract

**Background:**

Foot‐and‐mouth disease virus (FMDV) is an important pathogen of the MicroRNA virus family. Infection of livestock can cause physical weakness, weight loss, reduced milk production, and a significant reduction in productivity for an extended period. It also causes a high mortality rate in young animals, seriously affecting livestock production. The host range of FMDV is mainly limited to cloven‐hoofed animals such as cattle and sheep, while odd‐toed ungulates such as horses and donkeys have natural resistance to FMDV. The mechanism underlying this resistance in odd‐toed ungulates remains unclear.

**Objective:**

This study aimed to analyze the differences between FMDV‐infected cattle and horses to provide valuable insights into the host‐FMDV interaction mechanisms, thereby contributing to the control of foot‐and‐mouth disease and promoting the development of the livestock industry.

**Methods:**

We observed the distribution of integrins, which help FMDV enter host cells, in the nasopharyngeal tissues of cattle and horses using immunohistochemistry. Then, we employed high‐throughput RNA sequencing (RNA‐Seq) to study the changes in host gene expression in the nasopharyngeal epithelial tissues of cattle and horses after FMDV infection. We performed enrichment analysis of GO and KEGG pathways after FMDV infection and validated related genes through qPCR.

**Results:**

The immunohistochemical results showed that both cattle and horses had four integrin receptors that could assist FMDV entry into host cells. The transcriptome analysis revealed that after FMDV infection, pro‐apoptotic genes such as caspase‐3 (CASP3) and cytochrome C (CYCS) were upregulated in cattle, while apoptosis‐inhibiting genes such as NAIP and BCL2A1 were downregulated. In contrast, the expression trend of related genes in horses was opposite to that in cattle. Additionally, autophagy‐related genes such as beclin 1, ATG101, ATG4B, ATG4A, ATG13, and BCL2A1 were downregulated in cattle after FMDV infection, indicating that cattle did not clear the virus through autophagy. However, key autophagy genes including ATG1, ATG3, ATG9, ATG12, and ATG16L1 were significantly upregulated in horses after viral infection.

**Conclusion:**

Both water buffaloes and Mongolian horses express integrin receptors that allow FMDV entry into cells. Therefore, the resistance of Mongolian horses to FMDV may result from more changes in intracellular mechanisms, including processes such as autophagy and apoptosis. Significant differences were observed between water buffaloes and Mongolian horses in these processes, suggesting that these processes influence FMDV replication and synthesis.

## Graphical abstract

1. Both nonsusceptible animals (horses) and susceptible animals (cows) have integrin receptors in their nasopharyngeal tissues that can help FMDV enter cells.
2. Infection with the FMDV promotes apoptosis in cows, while the opposite is true for horses.
3. Autophagy‐related genes show a significant increase in expression in horses infected with FMDV.

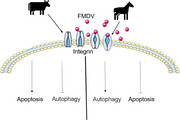

## 1 INTRODUCTION

Foot‐and‐mouth disease (FMD) is a highly contagious infectious disease that is prone to outbreaks in cattle farming. It is an acute febrile and highly transmissible disease caused by infection with the foot‐and‐mouth disease virus (FMDV). The typical clinical symptoms include high fever and the formation of numerous vesicles on the oral mucosa, hooves and udders (Alexandersen et al., 2003). As the disease progresses, the vesicles gradually rupture, and ulcers appear in the throat, bronchial tubes and stomach, resulting in haemorrhagic lesions. The FMDV rapidly replicates and spreads within the infected animal, between susceptible animals through contact, and through aerosols. The disease symptoms usually appear within 2–3 days after exposure and can last for 7–10 days (Grubman & Baxt, 2004). FMDV belongs to the Picornaviridae family, which is a positive‐sense RNA virus. It is a member of the Aphthovirus genus in the family, and there are currently seven serotypes of FMDV known: A, O, C, South African Territories (SAT) 1, SAT 2, SAT 3 and Asia 1. There is no antigenic cross‐protection between different serotypes, and new subtypes with altered antigenic characteristics often emerge due to the genetic instability of the virus (Belsham, 1993; Domingo, et al., 1995).

The emergence of new subtypes poses a significant challenge for the prevention and treatment of this disease. The FMDV genome consists of approximately 8400 nucleotides and contains one open reading frame (ORF), which encodes the viral polyprotein using the host cell's translation system. This polyprotein is cleaved to form four structural proteins (VP1, VP2, VP3 and VP4) and eight nonstructural proteins (Lpro, 2A, 2B, 2C, 3A, 3B, 3Cpro and 3Dpol) (Wang et al., 2010).

FMDV infection in livestock causes severe production losses and has a devastating impact on the economy of affected areas. Therefore, it is a major constraint for international animal trade, trade of animal products and economic cooperation between countries. This disease has occurred in almost all regions where livestock is raised. Over 100 countries worldwide are still affected by FMD, making it one of the most important diseases globally. The Office International Des Epizooties (OIE), also known as the World Organisation for Animal Health, and the Food and Agriculture Organization of the United Nations (FAO) classify FMD as one of the 18 most important A‐listed animal infectious diseases (Knight‐Jones & Rushton, 2013; Reddy & Golovkin, 2008). Therefore, conducting research on FMD is of significant importance for its control, prevention, eradication and minimising economic losses caused by the disease. As a highly contagious zoonotic disease, FMD primarily affects cloven‐hoofed animals, with some important domesticated animals and over 70 species of wild animals being susceptible to the virus. However, it is surprising and worth considering that odd‐toed ungulates, such as horses, are not susceptible to FMD and exhibit high resistance. Additionally, some carnivores also exhibit high resistance and do not show clinical symptoms or serotype characteristics even when contaminated with the virus. If contaminated animals come into close contact with susceptible livestock, they can also transfer the virus mechanically. Although the tropism of the foot‐and‐mouth disease (FMD) virus has been studied, the genetic mechanism of susceptibility to FMD remains largely unclear. There is still no relevant literature reporting on the high resistance of horses to FMD. Therefore, studying the differences in susceptibility between hosts for FMD is beneficial for elucidating the susceptibility mechanisms of this disease, developing effective preventative and antiviral drugs, and enhancing the control, prevention and eradication of FMD, ultimately reducing economic losses.

The transcriptome refers to the complete set of transcripts produced by a cell under specific conditions, such as different developmental stages or physiological states. It primarily focuses on the collection of all RNAs after transcription and their relative abundances. By analysing the expression of mRNA, researchers can study the expression characteristics of genes under various physiological conditions or in response to different environmental stimuli. Since the transcriptome is a dynamic process that changes with external signals, it can be altered as per the applied theory. Based on this concept, we can use transcriptome studies to explore the molecular mechanisms hidden behind a vast array of biological questions and pathological phenomena in humans, animals and plants. In recent years, there have been reports of studies using transcriptome analysis to study pathogens and their hosts, including research on plant disease resistance and analysis of microbial infections in animals. In fact, the scope of pathogens and hosts is very broad. When pathogens and hosts interact, their transcriptional products also change, allowing the application of transcriptomics to study these interactions and discover molecular mechanisms of their interactions, revealing the essence of diseases. In this study, transcriptomic technology was used to compare susceptible cattle and nonsusceptible horses, and bioinformatics analysis was performed to determine the expression levels of mRNA during infection with the FMDV, thereby revealing the function of key genes in response to viral infection. The aim was to elucidate the different genetic susceptibility mechanisms of Chinese Yellow Cattle and Mongolian Horses upon exposure to FMDV.

## 2 MATERIALS AND METHODS

### 2.1 Tissue culture and virus infection

The experimental samples of Yellow Cattle and Mongolian Horses were obtained from Inner Mongolia, China. After euthanising the animals, nasal and pharyngeal tissues were immediately collected from both species. The tissues were placed in PBS containing 100 UI/mL penicillin and 100 UI/mL streptomycin. The muscle tissue was removed from the nasal and pharyngeal regions, leaving only the mucosal epithelium. The nasal and pharyngeal mucosal epithelial tissues were cultured using the tissue block adherence method.

The O‐type Foot‐and‐Mouth Disease Virus strain (FMDV/O/HM/02) was preserved by the Inner Mongolia Beita Biotechnology Company in China. The successfully adhered tissue blocks were washed once with PBS. The maintenance medium (DMEM/F12 with 1% FBS(Gibco)) was mixed with the FMDV/O/HM/02 virus solution at a 1:1 ratio. The tissue blocks were then infected with the virus mixture for 24 h in a 37°C, 5% CO_2_ cell culture incubator.

### 2.2 Immunohistochemistry

Fix the nasal and pharyngeal tissues of Yellow Cattle and Mongolian Horses in 4% paraformaldehyde solution for 24 h. After fixation is complete, rinse with water for about 12 h. Dehydrate the tissues using a sucrose solution gradient, embed in OCT (liquid nitrogen slow freezing), and cut into sections using a cryostat microtome (thickness set at 5 µm). Wash the prepared tissue sections with PBS solution (pH = 7.4) for an extended period. Completely dehydrate the sections with methanol. Place in 0.5% H_2_O_2_ for 30 min, then wash once with distilled water. At room temperature, add fetal bovine serum to the tissue sections and incubate for 1 h. Remove the serum and add prediluted primary antibodies against Integrin β1, Integrin β3, Integrin β6 and Integrin β8 (sc‐8978, sc‐14009, sc‐15329, sc‐25714, Santa Cruz) to the sections and incubate at 4°C for about 20 h. Remove the primary antibodies, and at room temperature, add goat antirabbit IgG‐HRP (sc‐2004, Santa Cruz), incubate for 30 min. After incubation, wash the sections three times with PBS, for 5 min each. Perform DAB staining for 3 min, then wash with distilled water three times, finally stain the nuclei with haematoxylin. Positive staining appears brown‐yellow, negative staining appears blue. The negative control uses mouse serum as a substitute for the primary antibody.

### 2.3 RNA extraction, library construction and RNA‐Seq

The transcriptome sequencing library is based on the total RNA of the sample. However, in most biological organisms, rRNA accounts for the majority, with a content exceeding 90%, while mRNA content is only around 1–2%. If total RNA is directly used for sequencing, the results will inevitably be inaccurate. Therefore, it is necessary to purify the mRNA from the Total RNA for library construction. This process includes mRNA enrichment, fragmentation, reverse transcription of mRNA, ligation of adapters and PCR enrichment.

The experiment first collected normal control tissues and FMDV‐infected tissues from the nasopharynx of yellow cattle and Mongolian horses. The tissues were quickly ground into powder in liquid nitrogen, and then transferred to 1.5 mL centrifuge tubes with the addition of 1 mL TRIzol (Invitrogen, Life Thnics, CA, USA). After standing at room temperature for 5 min, the mixture was centrifuged at 12,000 rpm and 4°C for another 5 min. The supernatant was then aspirated and mixed with an equal volume of chloroform, shaken well and allowed to stand for 15 min. After centrifugation for 15 min, 400 µL of the supernatant was added to an equal amount of isopropanol, centrifuged again, and the resulting precipitate was washed with 75% ethanol. The final centrifugation precipitate was the total RNA of the sample. The purity and concentration of the RNA were then measured using a Nanodrop, agarose gel electrophoresis, and an Agilent 2100 bioanalyser to check for contamination, degradation and integrity of the RNA. After passing the quality inspection, enrichment of the mRNA from the sample was performed using oligo(dT)‐coupled magnetic beads. Then, fragmentation buffer was added to fragment the enriched mRNA, and complementary first‐strand cDNA was synthesised using the fragmented mRNA as a template. Second‐strand cDNA was synthesised by adding buffer, dNTPs and DNA polymerase I. The resulting double‐stranded cDNA was purified using AMPure XP beads. The purified double‐stranded cDNA was then subjected to PCR enrichment to obtain the cDNA library for subsequent sequencing.

The obtained cDNA library needs to be preliminarily quantified before it can be sequenced on the Hi Seq platform. We use Qubit 2.0 to quantify the library; dilute the cDNA library to a concentration of 1 ng/µL, and then detect the insert size using Agilent 2100. After the insert size meets the requirements, the effective concentration of the cDNA library needs to be quantified using the Q‐PCR method (the effective concentration should be >2 nM). Samples that meet the library quality requirements after these procedures will undergo Hi Seq sequencing.

### 2.4 Sequencing analysis process

After obtaining raw image data from the Illumina HiSeqTM2500 high‐throughput sequencing platform, the files need to be analysed for base recognition to convert them into Raw Reads (Lister et al., 2008), which contain both sequence information and corresponding quality information. The raw reads obtained from sequencing include many reads with unknown base information and low‐quality reads, so they cannot be directly used for subsequent analysis. Therefore, the sequencing data needs to be filtered through a model calculation formula that predicts the probability of base errors. The filtered clean reads are aligned to the reference genome using Tophat2, which is suitable for mammals. Cufflinks uses the Top Hat2 alignment results to assemble transcripts, yielding the smallest possible set of transcripts and enabling transcript quantification (Trapnell et al., 2012). Cuffdiff software is used to quantify mRNA, yielding FPKM information for each sample's transcripts. In RNA‐seq technology, FPKM represents the number of fragments per million fragments that are from a gene in every thousand base pairs length. This method of calculating gene expression levels is commonly used (Trapnell et al., 2010).

Using differential analysis, we can obtain transcripts that are significantly different between the experimental group and the control group, that is, the difference between virus‐infected and noninfected samples in this experiment. We can identify significantly differentially expressed transcripts between samples after virus treatment, which correspond to genes or target genes that play an important role in response to viral infection. The overall distribution of differentially expressed transcripts or genes can be visually displayed using a volcano plot, with a typical screening threshold set at *q*‐value < 0.05.

### 2.5 RNA reverse transcription and fluorescent quantitative PCR

Reverse transcribe cDNA using TAKARA Prime Script™ RT Master Mix (RR036A) and use NanoDrop to measure the cDNA concentration. Design primers for fluorescent quantitative PCR using primer3, a design tool provided by NCBI, with fragment sizes selected between 100–200 bp and other parameters set as default. Perform real‐time PCR using Roche LightCycler 480 II. Set the parameters as follows: preamplification: 95°C for 5 min; amplification: denaturation: 95°C for 20 s; annealing: 56–59°C (depending on the gene) for 20 s; extension: 72°C for 20 s; cycles: 40; collect fluorescence during the extension step; melting: 65°C for 5 s; cooling: 37°C for 5 s. Relative quantitative data analysis is performed using the 2^‐ΔΔCt^ method.

### 2.6 Statistical analysis

Differential gene expression was analysed using independent sample *t*‐test with SPSS 20.0 software, and GraphPad Prism 8.0 was used for graphing. The results are expressed as mean ± standard deviation. *p* < 0.05 indicates a significant difference; *p* < 0.01 indicates a highly significant difference.

## 3 RESULT

### 3.1 Expression of integrin receptors in the nasopharynx of cattle and horses

In the primary infection, the nasopharyngeal epithelial cells play a special role as a gateway for foot‐and‐mouth disease virus (FMDV). The persistent infection site of FMDV in cattle has been identified to be located in the nasopharyngeal mucosal epithelial cells, including the dorsal soft palate and the top of the nasopharynx (Pacheco et al., 2015; Stenfeldt et al., 2016). These areas have exposed active cells with appropriate receptors, making it easier for the virus to enter. After initial replication in the nasopharynx, FMDV enters the bloodstream and spreads to various parts of the body, causing a series of symptoms.

Integrins are the main receptors exploited by both enveloped and nonenveloped viruses for attachment and/or entry into cells. Integrins consist of two transmembrane subunits: α and β subunits. In mammals, 18 α and 8 β subunits combine in different combinations to form 24 integrins, which can bind to various extracellular matrix ligands (Stewart & Nemerow, 2007). FMDV attaches to host target cells (epithelial cells) through the interaction between the RGD motif on the viral capsid protein VP1 and integrin proteins on the cell surface, such as αvβ1, αvβ3, αvβ6 and αvβ8.

**FIGURE 1:**
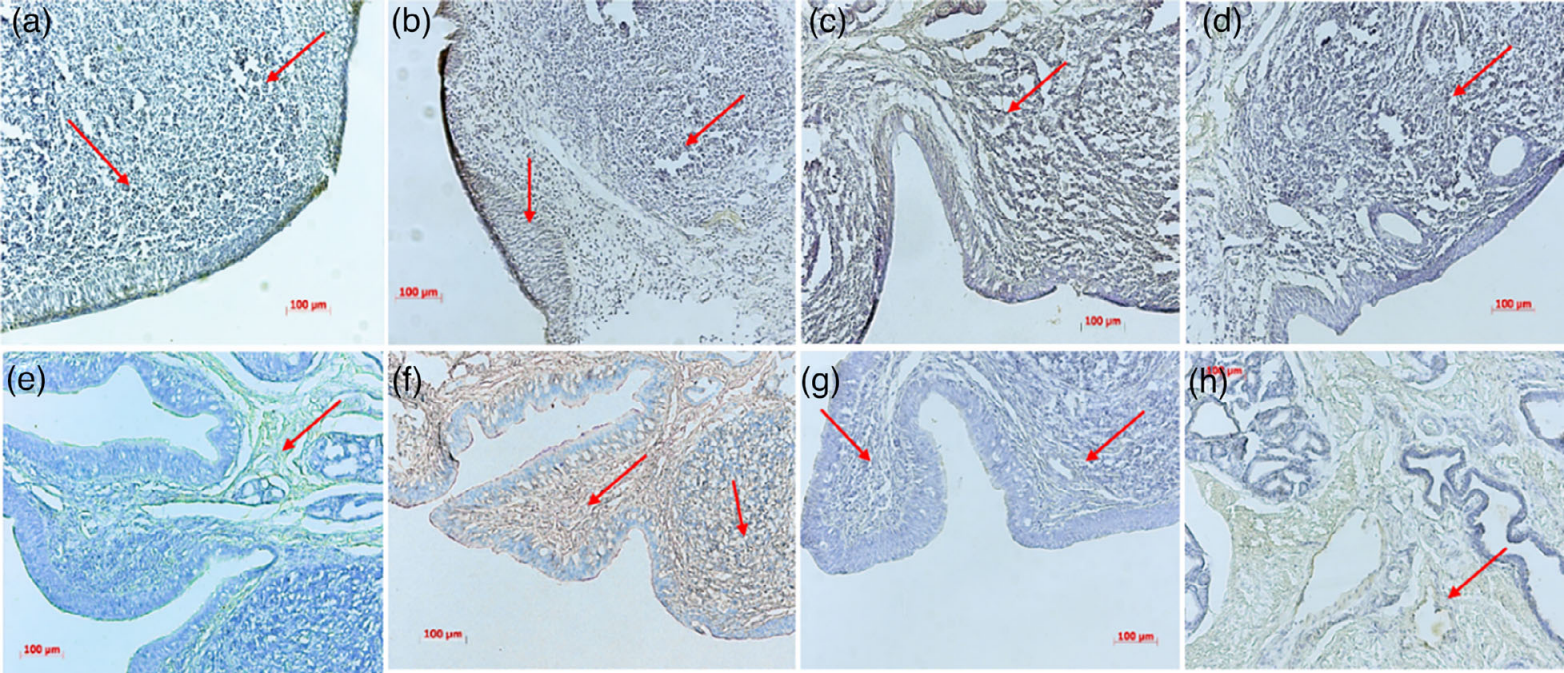
Integrin distribution of bovine and equine nasopharynx. (a) Bovine nasopharyngeal αvβ1 receptor. (b) Equine nasopharyngeal αvβ1 receptor. (c) Bovine nasopharyngeal αvβ3 receptor. (d) Equine nasopharyngeal αvβ3 receptor. (e) Bovine nasopharyngeal αvβ6 receptor. (f) Equine nasopharyngeal αvβ6 receptor. (g) Bovine nasopharyngeal αvβ8 receptor. (h) Equine nasopharyngeal αvβ8 receptor. The circled areas mark the integrin receptors.

Our results demonstrate that several confirmed FMDV receptors, including integrins αvβ1, αvβ3, αvβ6 and αvβ8, are expressed in the nasopharyngeal region of both cattle and horses. αvβ1 is abundant in the nasal mucosal epithelium of cattle, while it is less distributed in the intrinsic layer of the nasopharynx in horses. αvβ3 is distributed in the intrinsic layer of the nasopharynx in horses, but it shows negative staining in the mucosal epithelium. However, in cattle, αvβ3 is extensively expressed in both the intrinsic layer and the mucosal epithelium of the nasopharynx. αvβ6 shows significant differences in distribution between cattle and horses, with a higher distribution in the nasopharynx of cattle and a lower distribution in horses. The receptor αvβ8 is widely distributed in the nasopharyngeal region of both cattle and horses, with distribution observed on the outer side of the mucosal epithelium and the intrinsic layer (Figure 1). Therefore, it is possible that FMDV may enter horses through the same receptors as cattle, indicating that the difference in susceptibility to FMDV between even‐toed ungulates (such as cattle) and odd‐toed ungulates (such as horses) cannot be solely explained by the receptors (Figure 1).

### 3.2 Overall changes in gene expression after FMDV infection

Since the receptor‐mediated antiviral mechanism in horses could not be explained, this prompted us to conduct further research. We used transcriptome sequencing technology to study the susceptible site, hoping to elucidate the different mechanisms triggered by host cells in cattle and horses after infection with O‐type foot‐and‐mouth disease virus.

**FIGURE 2:**
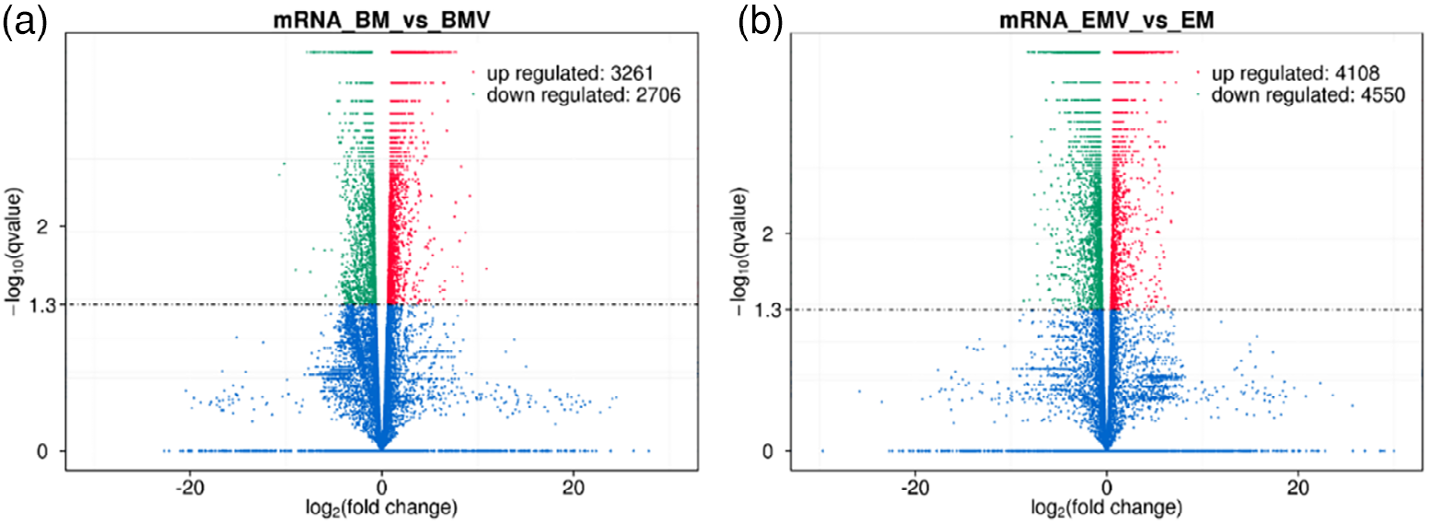
Volcano chart of bovine and equine nasopharynx after viral infection. (a) Volcano chart of bovine nasopharynx after viral infection. (b) Volcano chart of equine nasopharynx after viral infection. The *x*‐axis shows the log2(fold change) and *y*‐axis shows the‐log10(*q*‐value). Red dots represent the upregulated DEG, green dots represent the downregulated DEG and blue dots are the no significant difference genes.

After sequencing the samples, we used Cuffdiff software to perform differential comparison and analysis of the expression of the virus infection group and the normal control group in the pharynx of cattle and horses. Among them, the comparison between the virus infection group and the normal control group in cattle showed 5967 differentially expressed mRNAs, including 3261 upregulated genes and 2706 downregulated genes. The comparison between the virus infection group and the normal control group in horses showed a total of 8658 different mRNAs, including 4108 upregulated genes and 4550 downregulated genes. The volcano plot (Figure 2) intuitively shows the overall distribution pattern of gene expression. The screening threshold is set to *q*‐value < 0.05 by default.

### 3.3 GO and KEGG pathway analyses of DEG

We performed GO enrichment analysis using the ‘GOseq’ package in R, based on the Wallenius biased hypergeometric distribution, for both cattle and horses infected with FMDV. In the cattle dataset, we identified 5967 DEGs, while in the horse dataset, we identified 8658 DEGs. The analysis was conducted separately for the three branches of GO: Cellular Component (CC), Molecular Function (MF) and Biological Process (BP). A significance threshold of *p* < 0.01 was chosen to represent clustering significance.

**FIGURE 3:**
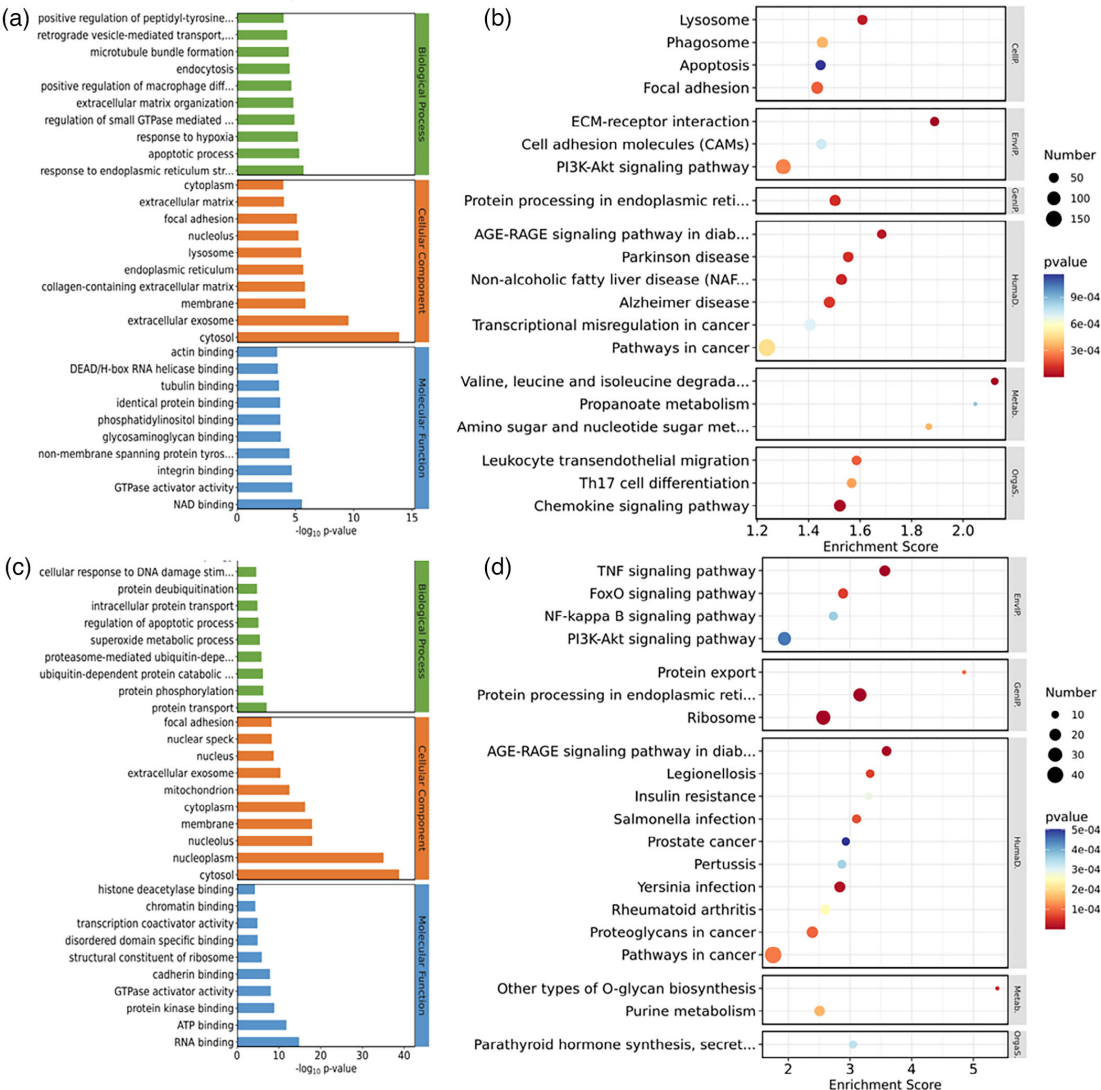
Transcriptome data show differences between bovine and *Equus caballus* after viral infection. (a) GO annotation of DEGs expressed in bovine. (b) Top 20 KEGG pathways of DEGs expressed in bovine. (c) GO annotation of DEGs expressed in *Equus caballus*. (d) Top 20 KEGG pathways of DEGs expressed in *Equus caballus*. GO terms were classified into three categories, including cellular component (CC), molecular function (MF), and biological process (BP). The GO terms were selected according to *p*‐value < 0.05.

In the cattle dataset, a total of 4997 GO terms were enriched, with 3275 terms in BP, 705 terms in CC and 1017 terms in MF (Figure 3a). On the other hand, in the horse dataset, there were 5115 enriched GO terms, with 3383 terms in BP, 717 terms in CC and 1015 terms in MF (Figure 3c). Furthermore, we performed KEGG pathway enrichment analysis. For cattle, we found a total of 168 significantly enriched pathways. Among these pathways, we selected the top 20 pathways with the most significant *p*‐values, which included pathways related to cell death, metabolism, and immune system (Figure 3b). For horses, we identified a total of 287 significantly enriched pathways. The top 20 pathways with the most significant *p*‐values included signalling pathways, protein translation and folding‐related pathways, as well as bacterial infection diseases (Figure 3d).

### 3.4 Differences in cell apoptosis between cattle and horses after FMDV infection

Apoptosis is a genetically programmed form of cell death that regulates the dynamic balance of the organism by eliminating inactive, injured or infected cells (Bertheloot et al., 2021). After being stimulated to undergo apoptosis, most cells exhibit mitochondrial outer membrane permeabilisation (MOMP) induced by proapoptotic members of the Bcl‐2 family, which allows for the release of cytochrome c from the mitochondria. Only proteins containing the BH3 domain(BAD, BID, BIK, BIM, BMF, HRK, NOXA, PUMA, etc.)can activate or control the relevant Bcl‐2 proteins (proapoptotic Bax and BAK) required for the formation of the complex structure necessary for MOMP, while antiapoptotic regulatory factors like Bcl‐2, Bcl‐xl and Mcl‐1 inhibit its formation (Kale et al., 2018). MOMP leads to the release of cytochrome c, which then activates apoptotic protease activating factor 1 (APAF‐1). Oligomerised cytochrome c‐APAF‐1 together with procaspase‐9 forms a large molecular complex called the apoptosome (Zhou et al., 2015). Subsequently, activated caspase‐9 from the apoptosome complex processes procaspase‐3 into active caspase‐3, which in turn degrades many substrates (Turpin et al., 2022).

**FIGURE 4:**
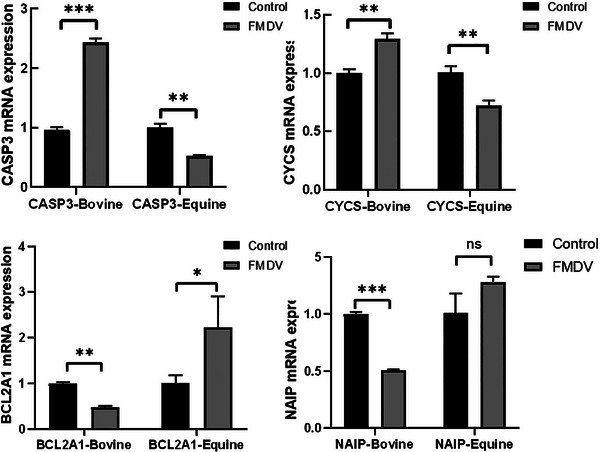
The expression of genes related to apoptosis after FMDV infection. *p < 0.05, **p < 0.01, ***p < 0.001; ns, not significant (two‐tailed unpaired Student's *t‐*test, compared to the control). Data are representative of three experiments with similar results.

We have found that after cattle are infected with the foot‐and‐mouth disease virus (FMDV), there is an upregulation in the expression of proapoptotic genes such as caspase 3 (CASP3) and cytochrome C (CYCS). Additionally, there is downregulation in the expression of apoptosis inhibiting genes like NAIP and BCL2A1 following infection. This suggests that FMDV negatively regulates antiapoptotic genes in cattle. Concurrently, FMDV enhances the expression of caspase‐3 and cytochrome C to further induce apoptosis. However, the related gene expression trends in horses are opposite to those in cattle (Figure 4). Recent studies have found that apoptotic caspases, particularly caspase‐3, can cleave cGAS, MAVS and IRF3, thereby effectively inhibiting the activation of innate immunity, while inhibiting apoptotic caspases significantly increases the production of I‐IFN in virus‐infected cells. Therefore, the apoptosis induced in cattle after FMDV infection may facilitate the virus in suppressing innate immunity, allowing for extensive viral replication. Horses, on the other hand, do not induce significant apoptosis during this process; moreover, the expression of apoptosis‐inhibitory genes could potentially enhance the production of I‐IFN, thus impeding viral replication.

### 3.5 Differences in autophagy between cattle and horses after FMDV infection

**FIGURE 5:**
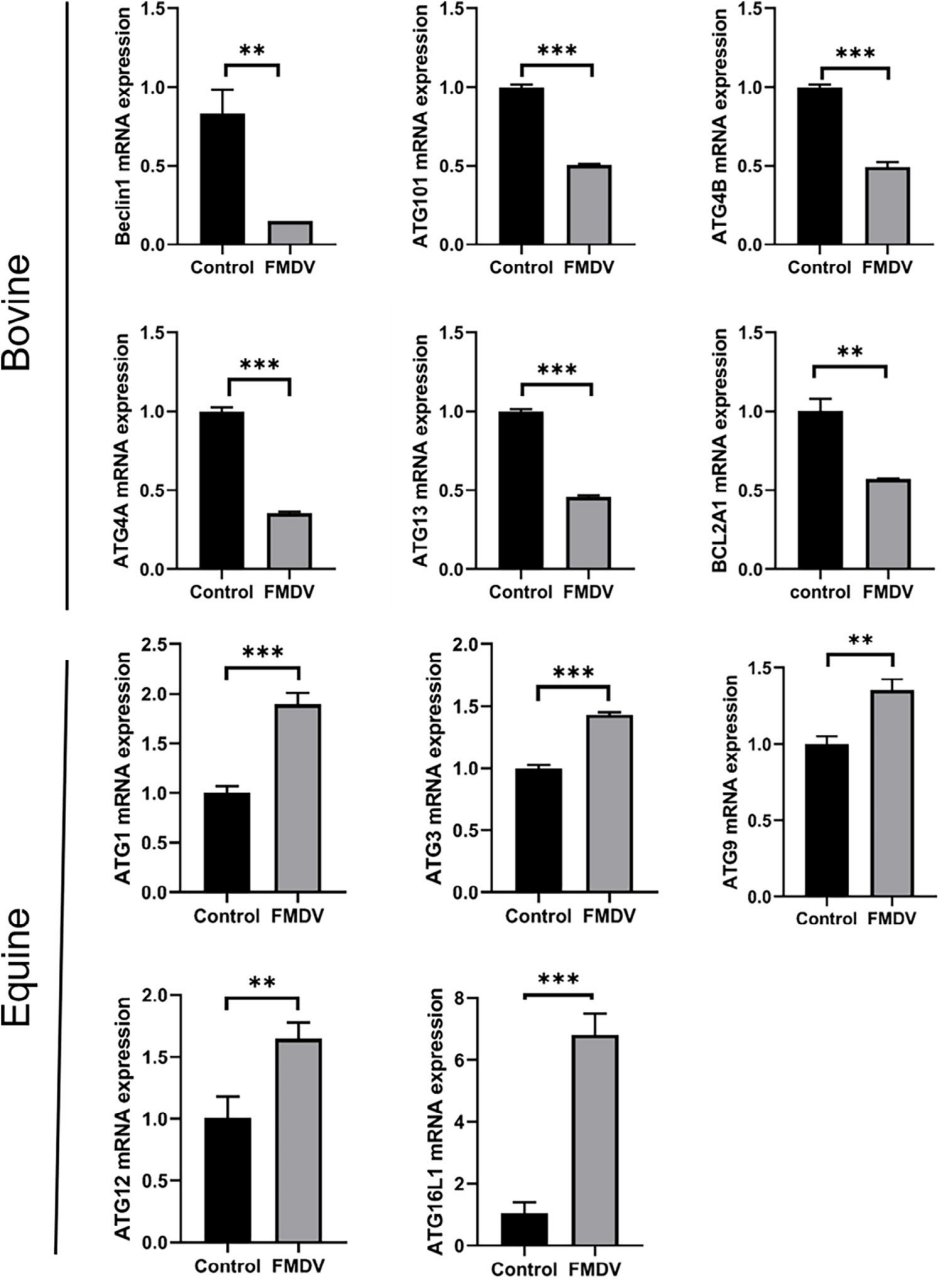
The expression of genes related to autophagy after FMDV infection in cattle. *p < 0.05, **p < 0.01, ***p < 0.001; ns, not significant (two‐tailed unpaired Student's *t*‐test, compared to the control). Data are representative of three experiments with similar results.

Differences in autophagy between cattle and horses after FMDV infection have also been observed. KEGG enrichment has linked autophagy pathways to cellular death‐related pathways including the lysosome, which is closely associated with autophagy. Like apoptosis, autophagy can act as a defence mechanism to eliminate pathogens. It starts with the pathogen signal being recognised by the pattern recognition receptor TLR3, which upregulates RIP1 through TRIF (Park et al., 2015), and then activates the autophagy pathway via ATG1. After autophagy clears the virus, it activates the lysosome and the ubiquitin‐proteasome degradation pathway. In cattle, the autophagy‐related genes beclin 1, ATG101, ATG4B, ATG4A, ATG13 and ATG16L2are all downregulated after FMDV infection, indicating that cattle do not clear the virus through autophagy. However, horses infected with FMDV, autophagy‐related genes such as ATG1, ATG3, ATG9, ATG12 and ATG16L1 are significantly upregulated, while the gene BCL2, which inhibits autophagy, is downregulated (Figure 5). The autophagy‐related gene 9 (ATG9) complex play an important role in the formation of autophagosomes as a source of membranes (Maiuri et al., 2007). Therefore, autophagy may play an important role in the clearance of FMDV in horses.

## 4 DISCUSSION

FMDV has a relatively strict tissue tropism, mainly infecting epithelial tissues in adult animals (Alexandersen et al., 2003). Integrins are heterodimers formed by the combination of α and β subunits, including 24 different αβ complexes formed by 18 different α subunits and 8 β subunits. It has been found that FMDV strains can use αvβ1, αvβ3, αvβ6 and αvβ8 integrins as receptors to invade cells (Wang et al., 2015). Through immunohistochemical experiments, we found that the four integrin receptors mentioned earlier were expressed in the nasopharyngeal tissues of both susceptible Holstein cattle and nonsusceptible Mongolian horses. In addition, transcriptome sequencing data showed that related integrin receptor genes, such as ITGA3, ITGA6, ITGA8, etc., were detected in both Holstein cattle and Mongolian horse datasets, and their expression was upregulated after FMDV infection. This suggests that there is no significant difference between Holstein cattle and Mongolian horses in the process of FMDV invasion, and therefore FMDV can enter Mongolian horse cells through the same receptors. The reason why FMDV does not further amplify and cause harm to Mongolian horses may be due to the cellular resistance mechanisms of the organism.

Immune evasion and cell apoptosis are both particularly important for effective viral infection and replication. However, given that caspases involved in apoptosis can inhibit innate immune signalling, while inhibiting apoptosis may lead to enhanced antiviral immune response, there is a dilemma between immune evasion and antiviral apoptosis. In this study, we found that cows exhibited enhanced cell apoptosis after FMDV infection, while apoptosis was inhibited in horses after FMDV infection. Viruses usually damage mitochondria by inducing swelling or other morphological changes, therefore, cGAS‐sting is activated by mitochondrial DNA(mtDNA) released in the cytoplasm. Upon detection of mtDNA, cGAS nucleotidyltransferase produces the second messenger cyclic GMP‐AMP (cGAMP), which binds to STING, leading to activation of TBK1, phosphorylation of IRF3 and production of IFN‐I(Uno & Ross, 2018). During viral infection, cGAS can be cleaved by apoptotic caspase‐3 (Ning et al., 2019), leading to the inactivation of cGAS catalytic activity and weakened cGAMP production (Fang & Peng, 2022). Additionally, the study found that FMDV infection can release a large amount of mtDNA, which reduces the expression of STING (Liu et al., 2023), indicating that the cGAS‐STING pathway is suppressed by caspase‐3. Meanwhile, during cell apoptosis, IRF3 can also be cleaved by caspase‐3 (Fang & Jiang et al., 2022). The analysis of interferon‐beta luciferase reporter showed that the cleaved fragment of IRF3 completely lost the ability to activate the interferon‐I promoter, so the increased apoptosis in cows after infection may inhibit the cGAS‐STING signalling pathway, thereby suppressing innate immune response and allowing FMDV to replicate extensively within cows. On the other hand, the situation is opposite in horses, which could be one of the reasons why horses do not exhibit symptoms of infection.

Autophagy maintains cellular homeostasis by degrading abnormal cytoplasmic components, damaged organelles, and invading pathogens targeted for degradation by lysosomes (Mizushima et al., 2008; Wirawan et al., 2012). Autophagy induction is regulated by autophagy‐related (ATG) proteins, which are recruited to membranes of various organelles to initiate autophagy (Biazik et al., 2015). As an important innate antiviral response, autophagy can be used to degrade viral components required for viral replication, viral particles and even host factors following viral infection. Several studies have also demonstrated the antiviral role of autophagy, as autophagy activation inhibited pseudorabies virus infection, while overexpression of autophagy‐related genes reduced Sindbis virus replication and virus‐induced mortality. Additionally, autophagy serves as a critical regulatory factor in innate immunity and controlling inflammasome activation (Zhou et al., 2022). In a mouse model infected with murine norovirus (MNV), interferon‐gamma‐mediated antiviral defence also requires the ATG5‐ATG12‐ATG16L1 complex, which functions in the formation of autophagosomes. In ATG16L1‐deficient mice, MNV infection leads to a phenotype resembling Crohn's disease (Choi et al., 2018). Cattle and horses exhibit different trends in changes of autophagy‐related genes following foot‐and‐mouth disease virus (FMDV) infection. Therefore, the role of autophagy in these two species is also different, with cattle tending to inhibit autophagy, preventing the degradation of viral particles and the efficient activation of innate immune pathways, while horses promote autophagy to exert antiviral effects.

## 5 CONCLUSION

It was commonly believed that odd‐toed ungulates, such as horses, were not infected with FMDV because they lacked integrin receptors on their cell membranes. However, based on the findings of this study, we discovered that both water buffalo and Mongolian horses express integrin receptors that allow FMDV to enter cells. Therefore, the resistance of Mongolian horses to FMDV may be due to more changes in intracellular mechanisms, including processes such as autophagy and apoptosis. In our data, there are significant differences between water buffalo and Mongolian horses in these two processes, indicating that these processes affect FMDV replication and synthesis. This discovery provides preliminary information for further research on the variation and epidemiological significance of FMDV pathogenesis.

## AUTHOR CONTRIBUTIONS

**Yi Wu**: Validation; writing—original draft. **Lu Li**: Formal analysis; methodology. **Wanfu Bai**: Data curation; investigation; software. **Tao Li**: Formal analysis; software. **Xiaoying Qian**: Resources; supervision. **Yiyi Liu**: Supervision. **Shenyuan Wang**: Formal analysis; investigation. **Chunxia Liu**: Resources; supervision. **Fang Wan**: Methodology. **Dong Zhang**: Conceptualisation. **Yingchun Liu**: Visualisation. **Kaifeng Wu**: Investigation. **Yu Ling**: Data curation; software; visualisation. **Huanmin Zhou**: Data curation; funding acquisition; investigation. **Fanhua Meng**: Supervision; writing—review and editing. **Yanru Zhang**: Investigation; methodology; project administration. **Junwei Cao**: Data curation; funding acquisition; project administration; resources.

## CONFLICT OF INTEREST STATEMENT

The authors declare no conflict of interest.

### ETHICS STATEMENT

The animal tissues used in this study were obtained from the North Asia Slaughterhouse in Hohhot, Inner Mongolia. The protocol for animal use was approved by the Ethics Committee of Inner Mongolia Agricultural University, with approval number NND2023109.

### PEER REVIEW

The peer review history for this article is available at https://publons.com/publon/10.1002/vms3.1569.

## Data Availability

The data that support the findings of this study are available from the corresponding author upon reasonable request.
